# Preclinical safety evaluation of *Macleaya Cordata* extract: A re-assessment of general toxicity and genotoxicity properties in rodents

**DOI:** 10.3389/fphar.2022.980918

**Published:** 2022-08-12

**Authors:** Zhen Dong, Shu-Sheng Tang, Xiao-Lan Ma, Chang-Hong Li, Zhao-Shan Tang, Zi-Hui Yang, Jian-Guo Zeng

**Affiliations:** ^1^ College of Veterinary Medicine, Hunan Agricultural University, Changsha, China; ^2^ Key Laboratory of Chinese Veterinary Medicine in Hunan Province, Hunan Agricultural University, Changsha, China; ^3^ College of Veterinary Medicine, China Agricultural University, Beijing, China; ^4^ College of Veterinary Medicine, Shanxi Agricultural University, Jinzhong, China; ^5^ Hunan MICOLTA Biological Resources Co., Ltd., Changsha, China

**Keywords:** *Macleaya cordata*, extract, rodent, acute toxicity, long-term toxicity, genotoxicity, teratogenicity

## Abstract

*Macleaya cordata* extract (MCE) is widely used for its diverse pharmacological actions and beneficial effects on farm animals. Modern pharmacological studies have shown that it has anti-inflammatory, anti-cancer, and anti-bacterial activities, and is gradually becoming a long-term additive veterinary drug used to improve animal intestinal health and growth performance. Although some evidence points to the DNA mutagenic potential of sanguinarine (SAN), a major component of MCE, there is a lack of sufficient basic toxicological information on the oral route, posing a potential safety risk for human consumption of food of animal origin. In this study, we assessed the acute oral toxicity, repeated 90-day oral toxicity and 180-day chronic toxicity of MCE in rats and mice and re-evaluated the genotoxicity of MCE using a standard combined *in vivo* and *ex vivo* assay. In the oral acute toxicity test, the LD_50_ for MCE in rats and mice was 1,564.55 mg/kg (95% confidence interval 1,386.97–1,764.95 mg/kg) and 1,024.33 mg/kg (95% confidence interval 964.27–1,087.30 mg/kg), respectively. The dose range tested had no significant effect on hematology, clinical chemistry, and histopathological findings in rodents in the long-term toxicity assessment. The results of the bacterial reverse mutation, sperm abnormality and micronucleus test showed negative results and lack of mutagenicity and teratogenicity; the results of the rat teratogenicity test showed no significant reproductive or embryotoxicity. The results indicate that MCE was safe in the dose range tested in this preclinical safety assessment. This study provides data to support the further development of maximum residue limits (MRLs) for MCE.

## Introduction

The *Papaveraceae* Juss. has about 38 genera and 700 species worldwide, mainly in the north temperate zone, especially in the Mediterranean region, West Asia, Central Asia to East Asia and southwestern North America (Zhang et al., 2020). Some of these species are used as medicines, especially *Corydalis yanhusuo* W. T. Wang in the genus *Corydalis* (Liu et al., 2021; Wu et al., 2021). However, a variety of plants in this family have obvious toxicity, such as *Papaver somniferum* L., *Macleaya cordata* (Willd.) R. Br., and *Chelidonium majus* L. Symptoms of poisoning manifest as depression of the central nervous system, liver damage, circulatory disorders, and gastrointestinal irritation (Moro et al., 2009; Günaydın et al., 2015; Lin et al., 2018). Because of its severe toxicity for human when taken orally, as documented in Tang Dynasty medical texts, *M. cordata* (Willd.) R. Br. (*M. cordata*) has been restricted to external use in China (Chen and Shang, 2002).

A variety of isoquinoline alkaloids have been identified in *M. cordata* (e.g., sanguinarine, dihydrosanguinarine, chelerythrine, dihydrochelerythrine, 6-cyano-dihydrochelerythrine, protopine, allocryptopine, berberine, etc.) (Ye et al., 2009; Qing et al., 2014, Qing et al., 2016a, Qing et al., 2016b; Lin et al., 2020), phenolic acids (ferulic acid, eugenol, p-hydroxybenzoic acid, and p-coumaric acid) and sterols (stigmasterol), etc. (Zou et al., 2016). Among them, alkaloids have the greatest advantage in terms of biological activity (Cai et al., 2020; Liu et al., 2022b).

Sanguinarine (SAN) and chelerythrine (CHE) are the most abundant alkaloids in *M. cordata*, which are also distributed in other plants of the poppy family, such as *Chelidonium majus* L., *Eomecon chionantha* Hance, etc. (Warowicka et al., 2019; Lu et al., 2020). They have a wide range of pharmacological activities, such as anti-inflammatory (Danielewski et al., 2022), anti-cancer (Zhu et al., 2020; Prabhu et al., 2021), antibacterial (Falchi et al., 2021; Lu et al., 2022), antifungal (Anjago et al., 2021), anthelmintic (Li et al., 2022), and immune enhancing (Liu et al., 2022a), etc. The biggest application of *M. cordata* is in livestock production, where studies have shown that MCE can provide a slight bitter taste in feed to increase animal intake (Chin, 2008; Wang et al., 2018). The main active ingredient of *M. cordata*, SAN, was included in the Chinese feed additive species list in 1999 (Ministry of Agriculture Announcement No. 105) and classified as a flavoring agent and spice management; SAN was approved as a flavoring agent by the European Union in 2004 and is on the feed additive list (Council of the European Union and European Parliament, 2008). Meanwhile, the standardized MCE product Sangrovit^®^ is approved for use in animal feeds as a plant-derived feed additive as an alternative to feeding growth-promoting antibiotics (AGPs) to promote animal growth and improve feed conversion (Juskiewicz et al., 2011). MCE is well tolerated in animals such as pigs, chickens, sheep and reindeer, and has been shown to significantly improve animal intestinal health, reduce oxidative stress damage, enhance innate immunity and disease resistance, and thus improve growth performance (Juskiewicz et al., 2011; Aguilar-Hernández et al., 2016; Chen et al., 2018; Chen et al., 2020; Bussabong et al., 2021). MCE does not induce the spread of antibiotic resistance genes and has a lower ecological risk (Zhang et al., 2021). In view of the obvious “antibiotic” nature of SAN, the 2008 edition of the “Feed Additive Species List” (Ministry of Agriculture Announcement No. 1126) issued by the Chinese Ministry of Agriculture and Rural Development removed SAN and transferred it to the veterinary medicine department for management. A growing body of research evidence demonstrating the benefits of MCE as a farm input for animal health and antibiotic resistance control has raised public and governmental concerns about the safety of MCE. Although some reports point out the risks associated with SAN, such as intraperitoneal injection of SAN can induce hepatotoxicity through oxidation of the protein thiol (Choy et al., 2008); In a zebrafish model, SAN can trigger cardiotoxicity by inducing extracellular Ca^2+^ influx and MAPK pathway (Hu et al., 2005; Wang et al., 2022); In addition, studies have shown the damaging effects of SAN on oocytes of mice fertilized *in vitro* and the increased embryonic resorption and reduced fetal weight after embryo transfer into the body (Chan, 2011, 2015). However, the concerns about toxicity of SAN were mainly attributed to the 1998 outbreak in New Delhi, India, of a disease known collectively as “epidemic dropsy” caused by contamination or adulteration of *Argemone mexicana* seed oil (AO) (Vaidya et al., 2001; Sharma et al., 2002). Numerous studies have shown evidence of a correlation between AO and genotoxicity (Ansari et al., 2006; Ghosh and Mukherjee, 2016). SAN is considered to be a major contributor to AO toxicity, and *in vitro* tests have shown that SAN can be embedded in DNA, and *in vivo* tests have shown the potential of SAN to damage DNA in blood and bone marrow cells in mice (Ansari et al., 2005). Zdarilova et al. (2008) evaluated the use of MCE as a feed additive in rats fed 600 mg per kg of feed for 90 days without any significant risk of organismal damage or genotoxicity (Stiborova et al., 2008). In addition, MCE was declared a veterinary drug for long-term use in China and its safety needs to be re-evaluated due to a change in use, with the EU removing MCE from the list of feed additives in May 2021 (Directorate-General for Health and Food Safety, 2021). Since the predominant route of exposure to exogenous compounds is oral, however, oral safety data for MCE are still vacant. In this study, we systematically evaluated the acute, long-term toxicity (repeated oral toxicity and chronic toxicity) of MCE in rodents. Given the risk of genotoxicity of SAN, we also conducted a series of standardized genetic toxicology tests, including the murine *Salmonella* revertant mutation (Ames) test, the mouse sperm aberration test, and the mouse bone marrow micronucleus test, to reassess the genotoxicity of MCE. In addition to this, a rat teratogenicity test was carried out to assess the embryotoxicity of MCE. Our findings provide data to support a comprehensive understanding of the potential risks of oral exposure to MCE.

## Materials and methods

### Test drugs and reagents

**FIGURE 1:**
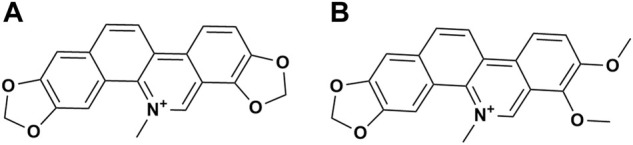
Chemical structure formula of the main compounds in MCE. **(A)** Sanguinarine; **(B)** Chelerythrine.

MCE, was provided by Hunan MICOLTA Biological Resources Co., Ltd. (Changsha, China). MCE is a standardized extract prepared by a series of processes, including heated sulfuric acid percolation, sodium hydroxide precipitation, filtration, drying, alcohol extraction, salt formation, and crushing. The total content of SAN (Figure 1A) and CHE (Figure 1B) in MCE is greater than 60%, with SAN ≥ 40% and CHE ≥ 20%, and the remaining trace components have been qualitatively characterized (Dong et al., 2021). The samples were kept at the Veterinary Drug Safety Supervision, Inspection and Testing Center of the Ministry of Agriculture and Rural Affairs, China Agricultural University (Beijing, China). Reagents for hematological and serum biochemical assays were purchased from Mindray Biomedical Electronics Co., Ltd. (Shenzhen, China). Other reagents were purchased from Sinopharm Chemical Reagent Co., Ltd. (Shanghai, China).

### Animals

SPF-grade SD rats and ICR mice were purchased from Beijing Vital River Laboratory Animal Technology Co., Ltd. (Beijing, China) and housed in a China Ministry of Agriculture and Rural Affairs certified GLP animal rearing room at the Veterinary Drug Safety Supervision, Inspection and Testing Center, Ministry of Agriculture and Rural Affairs, China Agricultural University, with controlled environmental parameters Conditions were as follows: temperature 25 ± 2°C, relative humidity 50% ± 20%, 12-h light/dark cycle, and ventilation 8–15 times/hour. All animals had free access to maintenance diets (crude protein ≥18%, crude fat ≥4%, crude fiber ≤5%, Beijing Keao Xieli Feed Co., Ltd.) and reverse osmosis water. All animal experiments were approved by the Animal Ethics Committee of China Agricultural University (Experiment approval number: WTPJ20090001-1∼7) and strictly followed the Chinese Guide for Ethical Review of Laboratory Animal Welfare (GB/T 35892, 2018).

### Oral acute toxicity in rats and mice

Sixty healthy SD rats (weighing 180–200 g) and ICR mice (18–22 g) were housed adaptively for 5 days. The oral acute toxicity test of MCE was carried out on rats and mice with reference to the half lethal dose method in the Compendium of Technical Guidelines for Veterinary Drug Research and the Guidelines for the acute toxicity (LD_50_ determination) of veterinary drugs (Ministry of Agriculture and Rural Affairs of PRC, 2009f; Veterinary Drug Assessment Centre of the Ministry of Agriculture of the and People’s Republic of China, 2012). The dose ranges were obtained from 2,500.00 to 879.20 mg/kg (rats) and 1750 to 573.44 mg/kg (mice) by pretesting, and the rats and mice were grouped separately (*n* = 10, half were males and the other half were females) using the complete randomization method, and the MCE was mixed in 1% CMCC-Na solution and transfused into rodents by oral gavage. The volume of the oral solution was 2 ml/100 g (rats) and 0.2 ml/100 g (mice) according to the protocol in Table 1. The rodents were observed for general behavior, signs of poisoning and mortality after poisoning.

**TABLE 1:** Oral acute toxicity dosing regimens in rats and mice.

Animals	Dose (mg/kg)	Animals	Dose (mg/kg)
Rats	2,500.00	Mice	1,750.00
Rats	2,000.00	Mice	1,400.00
Rats	1,600.00	Mice	1,120.00
Rats	1,280.00	Mice	896.00
Rats	1,024.00	Mice	716.80
Rats	819.20	Mice	573.44

### Repeated dose 90-day oral toxicity test in rats

Repeated Dose 90-Day Oral Toxicity test was conducted with reference to the Guidelines for 30-Day and 90-Day Feeding Tests for Veterinary Drugs (Ministry of Agriculture and Rural Affairs of PRC, 2009a). SD rats of 80–100 g (*n* = 20/group, half males, and the other half females) were divided into four groups and pellets were made at 156.46 (1/10 LD_50_), 31.49 (1/50 LD_50_), 6.26 (1/250 LD_50_) and 0 mg/kg of MCE added to the feed and given by *ad libitum* feeding for 90 days. At the end of 90 days, all surviving rats were anesthetized, heart blood was collected and executed.

### Chronic toxicity test in rats

The 180-day chronic toxicity test was conducted with reference to the Guidelines for Chronic Toxicity and Carcinogenicity Testing of Veterinary Drugs (Ministry of Agriculture and Rural Affairs of PRC, 2009c). Based on the results of 90-day subchronic toxicity, SD rats of 60–80 g (*n* = 50/group, half males, and the other half females) were divided into four groups and pellets were made at 78.23 (1/20 LD_50_), 15.65 (1/100 LD_50_), 3.13 (1/500 LD_50_) and 0 mg/kg of MCE added to the feed and given by *ad libitum* feeding for 180 days. At the end of 180 days, all surviving rats were anesthetized, heart blood was collected and executed.

### Hematology and serum biochemical tests

Blood from rats taken via the heart was tested separately in 90-day and 180-day long-term toxicity tests. Whole blood stored in vacuum EDTA-K2 anticoagulation tubes was examined on a BC-2800 Vet Animal Blood Cell Analyzer (Mindray Biomedical Electronics Co., Ltd., Shenzhen, China) for hemoglobin (Hg), red blood cell count (RBC), white blood cell count (WBC), and platelet count (PLT). Blood preserved in ordinary vacuum blood collection tubes without additives is separated by centrifugation to obtain serum. The serum alanine aminotransferase (ALT), aspartate aminotransferase (AST), urea nitrogen (BUN), creatinine (CR), glucose (GLU), albumin (ALB), total protein (TP), and total cholesterol (TCH) were measured using a BS-180 automated biochemical analyzer (Mindray Biomedical Electronics Co., Ltd., Shenzhen, China).

### Gross autopsy and histopathological examination

The major organs of rats anesthetized and executed in 90-day and 180-day long-term toxicity tests were removed from the surface blood and weighed after visual detection of tissue damage. All organs were preserved in 10% neutral buffered formalin solution for more than 3 days. The fixed tissues were washed with water, dehydrated in gradient ethanol, and treated with xylene before paraffin embedding. The prepared paraffin tissue block was sectioned and stained with hematoxylin & eosin (H&E) and then glued and stored. According to the requirements of veterinary drug declaration, if no toxicity-related histopathological changes are found, the relevant results are not required, so the histopathological picture results are not reflected in this report.

### *Salmonella typhimurium* revert mutation (Ames) test

Ames tests are conducted in accordance with the Guidelines for Ames Tests of Veterinary Drugs (Ministry of Agriculture and Rural Affairs of PRC, 2009b). Four test strains, TA97, TA98, TA100, and TA102, were tested by spot test and plate incorporation method using 100, 20, 4, 0.8, and 0.16 μg/ml of MCE, respectively. Additional solvent control, DMSO and positive control groups were set up. Positive drugs were selected as follows: 2 mg/ml of 2-aminofluorene (2-AF) was used for TA97, TA98, TA100, and TA102 for the point test plus S9. Without S9, TA97, TA98, TA100, and TA102 were all treated with 1 mg/ml of diquat. In the plate incorporation test, TA97, TA98, TA100, and TA102 used 100 μg/ml of 2-AF when S9 was added; TA97 used 50 μg/ml of acriflavine; TA98 used 200 μg/ml of 2,7-diaminofluorene (2,7-AF); TA100 used 15 μg/ml of sodium azide (NaN_3_) when S9 was not added; TA102 used 5 μg/ml of mitomycin; and TA97, TA98, TA100, and TA102 used 5 μg/ml of NaN_3_. TA100 used 15 μg/ml of NaN_3_; TA102 used 5 μg/ml of mitomycin C. The test was repeated twice and the number of revertant colonies formed in each test strain plate was counted after 48 h of incubation at 37°C. The test strains were purchased from China Veterinary Culture Collection Centre and approved for use by the Biosafety Committee of China Agricultural University.

### Mice bone marrow cell micronucleus test

Bone marrow cell micronucleus testing in mice according to the Guidelines for Bone Marrow Cell Micronucleus Testing in Veterinary Mice (Ministry of Agriculture and Rural Affairs of PRC, 2009g). ICR mice from 25 to 30 g were housed for 1 week for environmental adaptation. With reference to the results obtained for oral acute toxicity in mice, three MCE groups were set up in the test at concentrations of 512.17, 256.08, and 128.04 mg/kg, a positive control group (40 mg/kg, cyclophosphamide monohydrate, Reagent grade, 97.0%–103.0%, Beyotime Biotechnology Shanghai, China) and negative control group (distilled water, homemade) (*n* = 20, male and female). The drug was administered by gavage at 0.2 ml/10 g. The drug was administered twice with an interval of 24 h between doses. Mice were euthanized by cervical dislocation 6 h after the second administration, and bone marrow cells were harvested from mouse femurs using FBS, coated on slides, oven-dried, fixed in methanol, stained with Giemsa working solution (Beyotime Biotechnology, Shanghai, China) for 30 min, rinsed in distilled water, and air-dried for examination. The number of polychromatic erythrocytes (PCE), micronucleated polychromatic erythrocytes and mature erythrocytes (RBC) were counted by double-blind light microscopic examination. PCE micronucleus rate and PCE/RBC values were calculated for female and male mice in each test group, and the respective standard deviations were also calculated. The formula is as follows: PCE micronucleus rate = number of PCE containing micronucleus/number of PCE examinations × 100%; PCE/RBC ratio = number of PCE examinations/number of RBCs.

### Mice sperm abnormality test

Sperm deformity test for mice was conducted according to the Guidelines for Sperm Deformity Test for Veterinary Mice (Ministry of Agriculture and Rural Affairs of PRC, 2009e). Three MCE dose groups of 204.87 (1/5 LD_50_), 102.43 (1/10 LD_50_), and 51.22 mg/kg (1/20 LD_50_), a negative control group (distilled water, homemade) and a positive control group (40 mg/kg, cyclophosphamide, Reagent grade, 97.0%–103.0%, Beyotime Biotechnology, Shanghai, China) (*n* = 10, males). The drug was administered by gavage at 0.2 ml/10 g once a day for 5 days at 24 h intervals. On the 35th day after the first poisoning, the mice were executed by cervical dislocation and the epididymis was removed from both sides. Sperm solution was obtained using saline, and synthetic fiber blood mesh bags were filtered, and the filtrate was aspirated to make sperm smears. After air-drying, the smears were fixed with methanol for 10 min, dried and stained with 2% eosin (Solarbio Science & Technology Co., Ltd., Beijing, China) for 1 h, lightly rinsed with water and dried. Normal and abnormal spermatozoa (hookless, banana-shaped, fat-headed, amorphous, tail-folded, double-headed, double-tailed, etc.) were examined and counted under the microscope in the same field of view.

### Traditional teratogenicity test in rats

Referring to the Guidelines for Traditional Teratogenicity Tests on Veterinary Drugs in Rats with some modifications, we conducted a traditional teratogenicity test in rats (Ministry of Agriculture and Rural Affairs of PRC, 2009d) and made some modifications. Based on the results of oral acute toxicity tests in rats, 391.15 (1/4 LD_50_), 97.79 (1/16 LD_50_), and 24.45 mg/kg (1/64 LD_50_) and a negative control group (distilled water, homemade) were selected. Due to the instability of positive drug teratogenicity, a positive control group was not established for this test. The number of unmated young female rats in each group was 20 and the number of male rats was 10. Only female rats were used for gavage administration in the experiment, and males were used only for mating behavior. After the sexually mature male and female rats were caged together at 2:1, the females were checked daily to ensure that they had mated, and the mated females were used as the test rats, and the day of conception was taken as day zero. Repeated mating in the same cage was done until the number of rats detected that met the test conditions was sufficient for the test. The drug was administered from the 7th to the 16th day of conception, once/day for 10 consecutive days. The feeding, drinking and weight gain of pregnant rats were examined and recorded during the test period, and the general behavioral performance, poisoning and death of pregnant rats were observed. The pregnant rats were weighed on the 20th day of gestation, decapitated, and the ovaries and uterus were removed by dissection to check the number of corpus luteum, the number of absorbed fetuses, the number of stillbirths, the number of live fetuses, the male to female ratio of live fetuses, and the weight of ovaries and uterus. The live fetal rats were examined for weight, body length, tail length, and cosmetic abnormalities. Half of the live fetuses from each litter were fixed in 95% ethanol for 3 weeks, and the fixed fetuses were rinsed in water for several minutes and then placed in 2% potassium hydroxide solution for 72 h. The fetuses were then stained in alizarin red (sodium 3,4-dihydroxy anthraqinone-2-sulfonate) application solution (Beyotime Biotechnology, Shanghai, China) for 48 h and gently shaken twice a day until the skulls were stained red. The fetal rats were then removed and placed in transparent solution A (glycerol 20 ml, 2% potassium hydroxide solution 3 ml, add distilled water to 100 ml) for 2 days and in transparent solution B (glycerol 50 ml, 2% potassium hydroxide solution 3 ml, add distilled water to 100 ml) for 3 days, and the bones were left to stain red while the soft tissues were basically discolored. The stained fetal mouse specimens were observed under a stereomicroscope using a transmission light source, and then the skeleton was gradually examined for abnormalities. The other half of the fetal rats from each litter were placed in Bouin’s fluid (Solarbio Science & Technology Co., Ltd., Beijing, China) and fixed for 2 weeks for visceral examination.

### Statistical analysis

Data generated from the experiments were saved and organized by Microsoft Excel (Microsoft, Redmond, United States). Data were processed for appropriate statistical analysis such as *t* Test, One-way ANOVA and Chi-Squared Test using SPSS 19.0 (International Business Machines Corporation, Armonk, United States). Data are expressed as Mean ± SD and percentages. Image production was done by GraphPad prism 7.0 (GraphPad Software, San Diego, United States). *p*-values less than 0.05 and 0.01 were considered statistically significant and highly significant.

## Results

### Acute toxicity test in rats and mice

Acute oral toxicity tests were performed on SD rats and ICR mice, and the mortality of each dose group is shown in Table 2. The oral LD_50_ of MCE was calculated using Kärber’s method (Mantel, 1967) to be 1,564.55 mg/kg (95% confidence interval 1,386.97–1764.95 mg/kg) and 1,024.33 mg/kg (95% confidence interval 964.27–1,087.93) for rats and mice, respectively. mg/kg). Rats and mice showed signs of intoxication with erect back hair, arching of the back, and slow movement, and death occurred about 3 h after administration (Table 2). No obvious pathological organ damage was found at necropsy. Referring to the GHS acute toxicity classification criteria, MCE was found to be grade 4 (United Nations, 2015).

**TABLE 2:** Oral acute toxicity mortality in rats and mice at different doses of MCE.

Dosage (mg/kg)	Numbers	Number of deaths	Mortality rate (%)	Survival rate (%)
Rats				
2,500.00	10	10	100	0
2,000.00	10	7	70	30
1,600.00	10	5	50	50
1,280.00	10	3	30	70
1,024.00	10	1	10	90
819.20	10	0	0	100
1,750.00	10	10	100	0
Mice				
1,400.00	10	8	80	20
1,120.00	10	6	60	40
896.00	10	4	40	60
716.80	10	1	10	90
573.44	10	0	0	100

### Repeated 90-day oral toxicity test in rats

**FIGURE 2:**
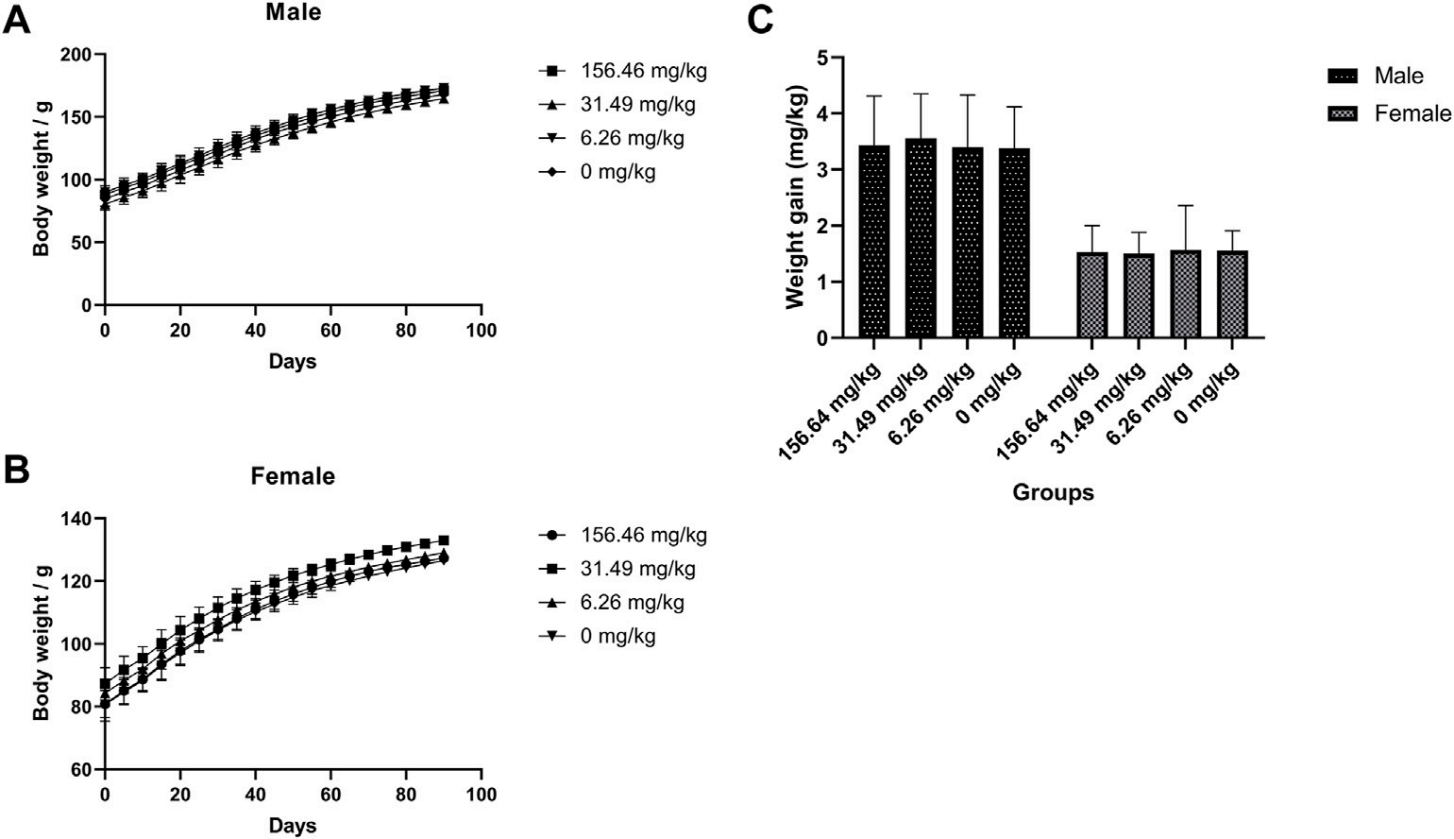
No effect of MCE on body weight gain in SD rats during 90 days of feeding. Male and female rats were orally administered 156.46, 31.49, 6.26, and 0 mg/kg (*n* = 10 rats/gender). **(A)** Body weight of male rats at 90 days; **(B)** Body weight of female rats at 90 days; **(C)** Body weight gain of male and female rats for 90 days.

The rats were given MCE at three doses of 156.46, 31.29, and 6.26 mg/kg for 90 days by *ad libitum* feeding, and the general behavior of the rats was normal during the observation period, with no significant differences in feeding, drinking and weight gain (Figure 2) compared to the blank control group (*p* > 0.05). There was no poisoning or death in each group. There were no significant differences in hematology (Table 3), serum biochemistry (Table 4), organ coefficients (Table 5), and histopathological examination in each test group compared with the control group (*p* > 0.05).

**TABLE 3:** Effect of feeding MCE for 90 and 180 days on hematological parameters in rats.

Groups	Hematological parameters (Mean ± SD)							
	Hg (g/L)		RBC (M/mm^3^)		WBC (th/mm^3^)		PLT (th/mm^3^)	
	M	F	M	F	M	F	M	F
90 days								
156.46 mg/kg	140.00 ± 4.34	140.40 ± 3.88	7.74 ± 0.15	7.26 ± 0.15	9.18 ± 0.71	7.64 ± 0.30	360.20 ± 29.05	351.00 ± 15.59
31.29 mg/kg	142.00 ± 2.83	145.20 ± 3.01	7.69 ± 0.20	7.14 ± 0.08	9.06 ± 0.48	7.36 ± 0.44	352.40 ± 40.29	344.60 ± 15.17
6.26 mg/kg	140.80 ± 3.43	145.40 ± 5.75	7.81 ± 0.38	7.31 ± 0.09	8.93 ± 1.18	7.58 ± 0.26	367.00 ± 29.46	360.00 ± 28.16
0.00 mg/kg	139.60 ± 2.73	139.40 ± 4.22	7.52 ± 0.21	6.99 ± 0.33	10.22 ± 1.23	7.86 ± 0.30	353.40 ± 16.27	350.20 ± 17.23
180 days								
78.23 mg/kg	140.40 ± 6.80	140.40 ± 6.80	6.44 ± 0.29	7.04 ± 1.49	10.85 ± 0.60	8.58 ± 0.33	245.60 ± 15.52	247.60 ± 12.62
15.65 mg/kg	140.40 ± 5.13	139.40 ± 3.36	6.66 ± 0.93	6.95 ± 0.22	10.01 ± 0.66	8.69 ± 0.42	244.80 ± 23.89	244.20 ± 8.70
3.13 mg/kg	141.60 ± 3.58	139.20 ± 4.49	6.61 ± 0.29	7.13 ± 0.17	10.02 ± 0.89	8.60 ± 0.74	245.40 ± 15.84	246.20 ± 12.19
0.00 mg/kg	141.00 ± 6.20	140.60 ± 4.83	6.63 ± 0.13	6.91 ± 0.48	10.52 ± 0.73	8.62 ± 0.89	248.00 ± 22.26	246.40 ± 17.33

Note: Hg, Hemoglobin; RBC, Red Blood Count; WBC, White Blood Count; PLT, Platelet Count.

**TABLE 4:** Effect of feeding MCE for 90 and 180 days on serum biochemical parameters in rats.

Serum biochemical parameters (Mean ± SD)																
Groups	ALT (U/L)		AST (U/L)		BUN (μmoL/L)		CR (μmoL/L)		GLU (mmoL/L)		ALB (mmoL/L)		TP (G/L)		TCH (mmoL/L)	
	M	F	M	F	M	F	M	F	M	F	M	F	M	F	M	F
90 days																
156.46 mg/kg	59.20 ± 3.71	59.20 ± 3.92	159.80 ± 3.71	157.40 ± 2.58	6.78 ± 0.17	7.68 ± 0.25	80.82 ± 4.04	89.12 ± 3.96	6.70 ± 0.55	6.22 ± 0.17	32.98 ± 1.35	39.40 ± 1.01	69.88 ± 3.84	78.74 ± 3.28	1.88 ± 0.16	1.94 ± 0.08
31.29 mg/kg	59.80 ± 3.54	60.80 ± 2.86	163.40 ± 6.72	158.40 ± 1.62	6.84 ± 0.65	7.56 ± 0.22	79.48 ± 2.72	90.32 ± 2.18	6.64 ± 0.41	6.24 ± 0.19	32.70 ± 1.30	38.30 ± 0.63	72.04 ± 4.06	78.81 ± 2.61	1.86 ± 0.10	1.82 ± 0.07
6.26 mg/kg	58.20 ± 2.32	59.00 ± 1.41	159.80 ± 5.64	158.60 ± 4.76	6.76 ± 0.60	7.58 ± 0.31	78.90 ± 3.25	90.44 ± 2.18	6.70 ± 0.32	6.32 ± 0.23	33.32 ± 1.14	38.22 ± 3.62	70.22 ± 2.31	78.20 ± 2.30	1.84 ± 0.10	1.86 ± 0.10
0.00 mg/kg	55.80 ± 5.91	60.00 ± 4.15	159.00 ± 5.73	158.40 ± 3.64	6.58 ± 0.40	7.62 ± 0.33	79.56 ± 4.16	91.58 ± 2.24	6.50 ± 0.58	6.34 ± 0.22	32.60 ± 1.31	39.50 ± 1.09	64.44 ± 14.19	79.50 ± 2.77	1.86 ± 0.30	2.04 ± 0.12
180 days																
78.23 mg/kg	58.20 ± 4.21	73.40 ± 3.21	173.20 ± 3.42	188.60 ± 1.14	8.86 ± 0.37	9.72 ± 0.45	76.70 ± 4.43	74.80 ± 2.16	7.52 ± 0.59	8.04 ± 0.18	32.88 ± 1.14	27.72 ± 1.13	72.28 ± 0.64	68.30 ± 0.71	1.52 ± 0.08	1.44 ± 0.15
15.65 mg/kg	58.40 ± 4.72	72.60 ± 4.51	173.40 ± 3.58	189.20 ± 5.07	8.94 ± 0.59	9.71 ± 0.14	77.82 ± 2.34	75.00 ± 0.71	7.56 ± 0.42	7.94 ± 0.52	32.42 ± 2.49	27.38 ± 0.92	73.06 ± 1.80	68.84 ± 2.04	1.60 ± 0.07	1.42 ± 0.11
3.13 mg/kg	58.00 ± 1.87	73.20 ± 3.11	173.00 ± 3.39	188.20 ± 5.07	8.92 ± 0.37	9.78 ± 0.25	77.06 ± 2.00	74.66 ± 1.67	7.50 ± 0.12	8.20 ± 0.59	32.60 ± 1.15	27.94 ± 0.34	72.96 ± 0.79	68.92 ± 1.64	1.58 ± 0.08	1.42 ± 0.08
0.00 mg/kg	58.20 ± 2.28	72.40 ± 8.91	173.20 ± 2.41	185.00 ± 6.11	8.82 ± 0.59	9.76 ± 0.27	77.30 ± 3.14	74.46 ± 1.56	7.54 ± 0.43	7.98 ± 0.45	32.74 ± 0.42	27.44 ± 1.13	77.92 ± 4.02	68.80 ± 1.30	1.58 ± 0.08	1.46 ± 0.11

Note: ALT, Alanine aminotransferase; AST, Aspartate aminotransferase; BUN, Blood urea nitrogen; CR, Creatinine; GLU, Glucose; ALB, Albumin; TP, Total protein; TCH, Total cholesterol.

**TABLE 5:** Effect of feeding MCE for 90 and 180 days on organ coefficients in rats.

Groups	Liver		Kidney		Spleen		Stomach and intestines		Lung		Heart		Testicles	Ovaries
	M	F	M	F	M	F	M	F	M	F	M	F		
156.46 mg/kg	3.22 ± 0.10	3.80 ± 0.12	0.64 ± 0.04	0.69 ± 0.06	0.14 ± 0.01	0.21 ± 0.02	6.74 ± 0.41	8.89 ± 0.57	0.46 ± 0.03	0.72 ± 0.06	0.31 ± 0.01	0.34 ± 0.04	0.64 ± 0.02	0.04 ± 0.01
31.29 mg/kg	2.98 ± 0.28	3.83 ± 0.13	0.65 ± 0.03	0.73 ± 0.04	0.13 ± 0.01	0.21 ± 0.01	6.21 ± 0.63	9.00 ± 0.35	0.46 ± 0.03	0.72 ± 0.03	0.30 ± 0.02	0.35 ± 0.02	0.64 ± 0.02	0.04 ± 0.00
6.26 mg/kg	3.19 ± 0.06	3.86 ± 0.08	0.66 ± 0.02	0.71 ± 0.01	0.13 ± 0.01	0.21 ± 0.01	6.35 ± 0.91	8.69 ± 0.33	0.46 ± 0.01	0.65 ± 0.16	0.30 ± 0.01	0.35 ± 0.02	0.63 ± 0.01	0.04 ± 0.00
0.00 mg/kg	3.21 ± 0.08	3.76 ± 0.12	0.64 ± 0.04	0.69 ± 0.07	0.14 ± 0.01	0.21 ± 0.02	6.73 ± 0.37	8.81 ± 0.60	0.46 ± 0.03	0.69 ± 0.05	0.31 ± 0.01	0.34 ± 0.01	0.64 ± 0.02	0.04 ± 0.00
78.23 mg/kg	3.05 ± 0.19	3.02 ± 0.19	0.56 ± 0.02	0.56 ± 0.05	0.15 ± 0.01	0.17 ± 0.01	6.34 ± 0.31	7.02 ± 1.40	0.45 ± 0.03	0.62 ± 0.01	0.26 ± 0.03	0.24 ± 0.01	0.61 ± 0.01	0.03 ± 0.01
15.65 mg/kg	2.92 ± 0.11	2.99 ± 0.08	0.59 ± 0.04	0.59 ± 0.04	0.15 ± 0.02	0.17 ± 0.01	6.20 ± 0.30	6.94 ± 0.34	0.44 ± 0.02	0.62 ± 0.02	0.28 ± 0.02	0.25 ± 0.01	0.59 ± 0.04	0.03 ± 0.00
3.13 mg/kg	3.06 ± 0.09	2.85 ± 0.10	0.62 ± 0.07	0.68 ± 0.01	0.14 ± 0.02	0.15 ± 0.01	6.40 ± 0.39	7.61 ± 0.74	0.59 ± 0.05	0.60 ± 0.11	0.26 ± 0.03	0.29 ± 0.03	0.59 ± 0.05	0.03 ± 0.00
0.00 mg/kg	3.00 ± 0.05	2.91 ± 0.07	0.57 ± 0.01	0.59 ± 001	0.15 ± 0.01	0.16 ± 0.01	6.12 ± 0.25	7.14 ± 0.38	0.44 ± 0.02	0.61 ± 0.01	0.26 ± 0.00	0.24 ± 0.02	0.61 ± 0.02	0.02 ± 0.00

Note: Organ coefficient = (organ weight/body weight) x 100%.

### Chronic toxicity in rats

**FIGURE 3:**
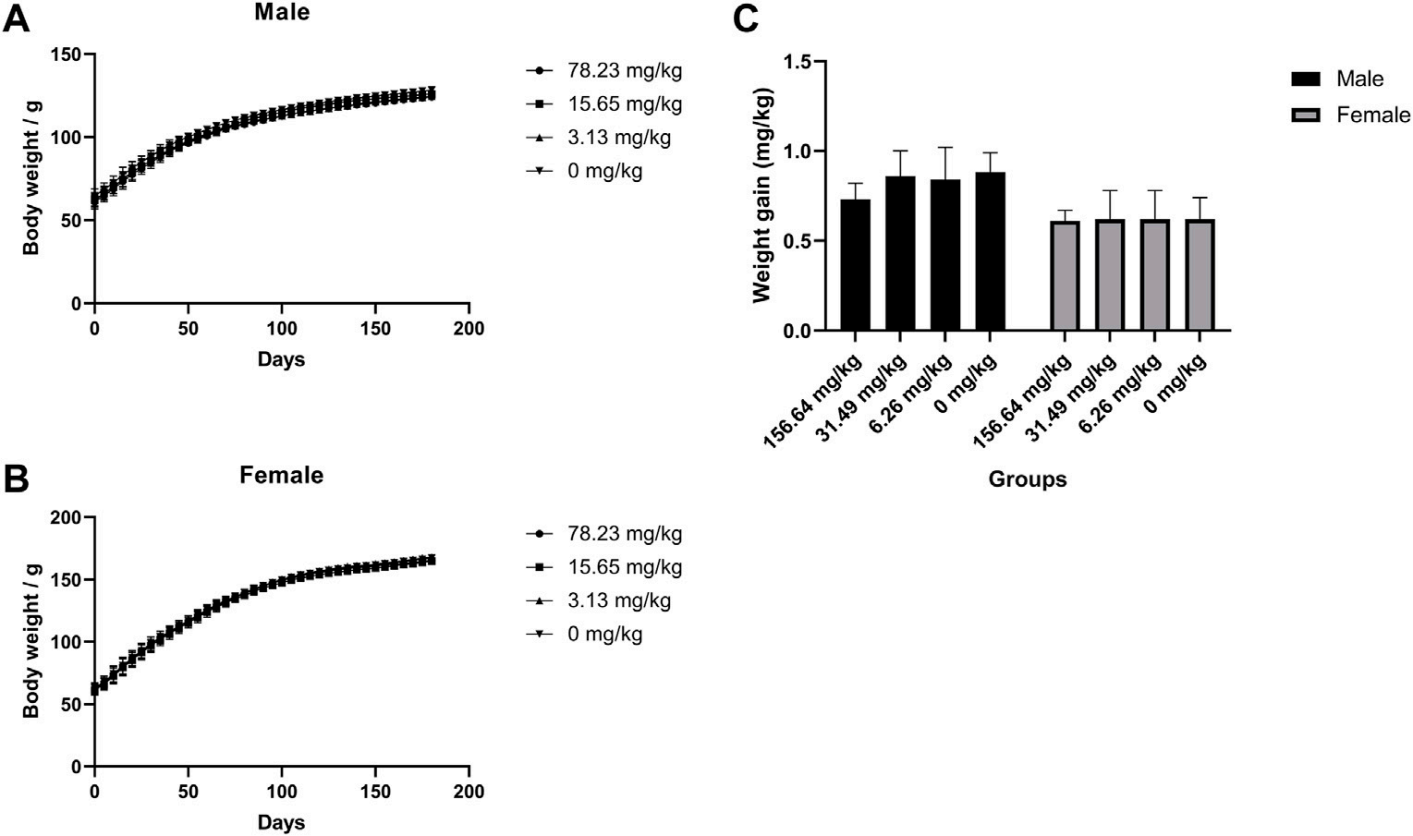
No effect of MCE on body weight gain in SD rats during 180 days of feeding. Male and female rats were orally administered 78.23, 15.65, 3.13, and 0 mg/kg (*n* = 25 rats/gender). **(A)** Body weight of male rats at 180 days; **(B)** Body weight of female rats at 180 days; **(C)** Body weight gain of male and female rats for 180 days.

The potential chronic toxicity risk is of particular concern because MCE has been added to feed for long periods of time for clinical use. The rats were fed at 78.23, 15.65, and 3.13 mg/kg for 180 days and did not exhibit any significant abnormalities or die during the observation period in all dose groups. Similarly, MCE did not produce a significant effect (*p* > 0.05) on the desire to eat and drink and weight gain of the rats (Figure 3). Further hematological (Table 3), serum biochemical (Table 4), organ coefficients (Table 5) and histopathological examinations also did not observe significant changes compared to the control group (*p* > 0.05).

### Ames test

Four test strains of *S. typhimurium* grew normally, and each strain in the positive control group reacted satisfactorily. MCE was in the range of 100–0.16 μg/dish, and the mean number of revertant colonies per dish for all four test bacteria in the presence or absence of the metabolic activation system (S9) was within twice that of the solvent (sterilized distilled water) control, and no dose-response relationship was seen, and the results of the two tests were consistent. It was shown that MCE was not mutagenic to *S. typhimurium* for the test. The results of the two replicate tests are shown in Table 6.

**TABLE 6:** Ames test results for MCE (Mean ± SD).

Groups	TA_97_		TA_98_		TA_100_		TA_102_	
	−S9	+S9	−S9	+S9	−S9	+S9	−S9	+S9
100 μg/ml	123.7 ± 10.7	131.7 ± 3.5	34.3 ± 2.5	32.0 ± 1.0	151.3 ± 1.5	160.0 ± 6.2	269.0 ± 8.5	252.3 ± 8.5
	125.3 ± 5.1	130.0 ± 7.7	40.0 ± 4.6	41.0 ± 2.6	159.7 ± 8.5	171.7 ± 6.1	285.3 ± 9.5	262.7 ± 4.7
20 μg/ml	126.7 ± 5.5	134.3 ± 10.7	36.0 ± 3.6	39.0 ± 2.6	156.7 ± 2.1	157.7 ± 5.5	279.3 ± 7.4	262.0 ± 6.1
	130.7 ± 13.3	129.7 ± 2.3	44.7 ± 5.9	41.0 ± 3.6	167.0 ± 2.6	166.3 ± 10.3	281.7 ± 14.5	274.0 ± 7.9
4 μg/ml	120.3 ± 10.0	127.7 ± 5.5	32.3 ± 2.1	34.7 ± 4.0	154.3 ± 1.5	146.0 ± 2.6	264.3 ± 4.2	250.0 ± 2.6
	131.7 ± 11.6	143.7 ± 10.7	39.7 ± 8.1	41.7 ± 4.6	162.0 ± 8.7	161.7 ± 6.8	283.7 ± 17.4	262.7 ± 17.0
0.8 μg/ml	113.7 ± 9.3	138.3 ± 2.1	36.3 ± 2.3	36.0 ± 3.0	166.3 ± 3.8	147.0 ± 1.7	288.3 ± 6.0	246.3 ± 4.2
	120.3 ± 6.5	122.0 ± 4.4	37.0 ± 6.6	42.0 ± 6.6	170.7 ± 18.1	159.0 ± 7.0	280.7 ± 3.5	271.0 ± 1.0
0.16 μg/ml	137.7 ± 4.5	126.0 ± 11.0	39.0 ± 2.0	40.7 ± 1.2	160.7 ± 4.0	148.7 ± 3.5	272.3 ± 5.0	260.3 ± 5.0
	123.3 ± 6.0	132.3 ± 8.4	38.7 ± 7.1	41.3 ± 6.1	166.0 ± 9.5	159.0 ± 7.0	280.7 ± 3.5	271.0 ± 1.0
Solvent control	129.0 ± 9.6	127.0 ± 7.5	33.3 ± 1.5	33.3 ± 3.1	160.3 ± 6.8	145.3 ± 5.7	275.3 ± 10.5	261.3 ± 6.7
	124.3 ± 5.1	127.0 ± 13.5±	40.3 ± 7.5	40.0 ± 6.2	170.7 ± 8.7	156.0 ± 3.5	272.0 ± 1.0	273.3 ± 7.2
DMSO	132.0 ± 9.6	129.7 ± 4.9	37.0 ± 2.0	33.3 ± 2.1	149.0 ± 3.6	162.7 ± 6.0	279.7 ± 5.1	259.0 ± 8.7
	136.0 ± 9.5	132.3 ± 3.8	42.0 ± 2.6	37.7 ± 5.1	165.0 ± 4.4	172.0 ± 10.4	270.3 ± 10.7	271.3 ± 6.7
Positive control	579.3 ± 14.0	874.7 ± 15.0	283.7 ± 13.2	560.7 ± 10.0	758.0 ± 12.2	779.7 ± 12.2	1,148.0 ± 20.5	932.3 ± 23.2
	564.3 ± 12.3	873.3 ± 13.3	564.3 ± 12.3	569.0 ± 14.5	564.3 ± 12.3	795.7 ± 6.4	1,197.7 ± 49.5	935.7 ± 8.5
	Acriflavine (5 μg)	2-AF (10 μg)	2,7-AF (20 μg)	2-AF (10 μg)	NaN_3_ (1.5 μg)	2-AF (10 μg)	MMC (0.5 μg)	2-AF (10 μg)

Note: NaN_3_—Sodium azide; 2-AF, 2-Aminofluorene; 2,7-AF, 2,7-Diaminofluorene; MMC, Mitomycin C.

### Mouse bone marrow cell micronucleus test

Due to the solid evidence of the reported mutagenic effect of SAN on bone marrow cell DNA *in vivo*, we conducted a mouse bone marrow cell micronucleus assay. The results showed that the positive control group (cyclophosphamide) contained a significantly higher rate of micronucleated PCEs than the negative control group (*p* < 0.01), in contrast, the values of PCE/RBC were within the normal range in all dose groups. The PCE rates of bone marrow cells in female and male mice in each dose group of MCE were not significantly different from solvent controls (*p* > 0.05), indicating the lack of mutagenicity of MCE in mice (Table 7).

**TABLE 7:** Results of the micronucleus test in mouse bone marrow cells with MCE.

Groups	PCE/RBC		PCE micronucleus rate (%)	
	F (*n* = 5)	M (*n* = 5)	F (*n* = 5)	M (*n* = 5)
512.17 mg/kg	1.19 ± 0.21	0.96 ± 0.09	1.40 ± 0.56	1.20 ± 0.24
256.08 mg/kg	1.02 ± 0.43	1.00 ± 0.14	1.00 ± 0.20	1.19 ± 0.08
128.04 mg/kg	1.03 ± 0.32	1.09 ± 0.28	1.40 ± 0.40	1.60 ± 0.42
Negative control	1.10 ± 0.38	1.06 ± 0.14	1.20 ± 0.62	1.19 ± 0.09
CTX	1.30 ± 0.79	1.58 ± 0.49	22.60 ± 1.87*	17.8 ± 2.35*

Note: CTX, Cyclophosphamide; * indicates *p* < 0.05 compared to negative control group.

### Mice sperm abnormality test

Subsequently, we tested whether MCE can cause sperm cell aberrations *in vivo* using a mouse sperm deformation assay. The sperm malformation rate of mice in each dose group of MCE did not show significant differences from the negative control group after five doses of exposure. In contrast to the significant increase in sperm malformations caused by cyclophosphamide-treated group, sperm abnormality rates in mice remained essentially unchanged at any of the MCE doses tested (Table 8), indicating that MCE is not teratogenic in mice.

**TABLE 8:** Sperm abnormality test for MCE in mice.

Groups	Number of animals	Sperm count	Abnormal sperm count	Sperm abnormality rate (%)	Percentage of deformed sperm species (%)							
					No hook	Banana-shaped	Amorphism	Fat head	Folded tail	Double heads	Double tails	Other
204.87 mg/kg	5	5 × 1,000	130	2.60	35.38	3.08	30.77	16.92	12.31	1.54	0	0
102.43 mg/kg	5	5 × 1,000	132	2.64	31.82	3.79	34.85	13.64	14.39	1.52	0	0
51.22 mg/kg	5	5 × 1,000	126	2.52	30.16	4.76	31.06	14.29	12.07	3.97	1.59	0
Negative control	5	5 × 1,000	138	2.56	20.88	8.09	28.99	13.04	20.29	5.07	2.17	0
Positive control	5	5 × 1,000	495*	9.90*	38.99	3.06	40.81	7.27	1.82	3.84	1.21	0

Note: * indicates *p* < 0.05 compared to negative control group.

### Traditional teratogenicity test in rats

Because of the previously reported risk of embryotoxicity of SAN (Chan, 2015), we further investigated the potential embryotoxicity of MCE using a conventional teratogenic assay in rats. The results showed that MCE did not produce maternal toxicity in pregnant rats in the dose range of 24.45–391.15 mg/kg, and the pregnant rats showed normal behavior, no poisoning and death, and no significant differences in drinking, feeding and weight gain compared with the negative control group (*p* > 0.05) (Table 9). There was no significant difference in ovarian weight, number of corpus luteum, number of implantations, uterine weight and number of live fetuses between the dose groups compared to the negative control group (*p* > 0.05) (Table 9). No teratogenic effect of MCE on fetal rats was also found in the examination of fetal rats (stillbirth, appearance, viscera, and bones) (Tables 10, 11). The test results showed that MCE had no significant reproductive toxicity or embryotoxicity to rats.

**TABLE 9:** Effect of MCE on the reproductive performance of pregnant rats.

Groups	Number of pregnant rats	Weight gain	Ovarian weight	Number of corpus luteum	Number of nidation	Uterine weight	Average number of live births
391.15 mg/kg	12	2.47 ± 0.29	0.13 ± 0.01	16.33 ± 0.82	187	5.12 ± 0.17	15.50
97.79 mg/kg	12	2.59 ± 0.34	0.13 ± 0.01	16.50 ± 0.92	194	5.27 ± 0.18	16.08
24.45 mg/kg	12	2.15 ± 0.37	0.12 ± 0.01	16.42 ± 0.85	191	5.21 ± 0.13	15.75
Negative control	12	2.13 ± 0.36	0.12 ± 0.01	15.92 ± 0.71	185	5.18 ± 0.14	15.25

**TABLE 10:** Teratogenic effects of MCE in pregnant rats.

Groups	Number of pregnant rats	Malformed appearance			Malformed bones			Malformed viscera		
		Number of fetal rats examined	The incidence of malformed fetuses (%)	Maternal malformation rate (%)	Number of fetal rats examined	The incidence of malformed fetuses (%)	Maternal malformation rate (%)	Number of fetal rats examined	The incidence of malformed fetuses (%)	Maternal malformation rate (%)
391.15 mg/kg	12	186	0	0	96	5.20	33.33	90	0	0
97.79 mg/kg	12	193	0	0	95	7.37	50.00	98	0	0
24.45 mg/kg	12	189	0	0	997	6.18	41.67	92	0	0
Negative control	12	183	0	0	94	7.44	50.00	89	0	0

Note: The incidence of malformed fetuses (%) = (numbers of malformed fetuses/numbers of fetal rats examined) × 100%; maternal malformation rate (%) = (numbers of pregnant rats with malformed fetuses/numbers of pregnant rats examined) × 100%.

**TABLE 11:** Effect of MCE on fetal rats.

Groups	Fetal resorption rate (%)	Fetal death rate (%)	Live fetus rate (%)	Female/male ratio	Average placental weight	Average fetal rat weight	Average body length of fetal rats(cm)	Average tail length of fetal rats (cm)
391.15 mg/kg	0.53	0	99.47	91/95	0.45 ± 0.26	3.65 ± 0.23	3.55 ± 0.06	1.13 ± 0.04
97.79 mg/kg	0.52	0	99.48	91/102	0.43 ± 0.02	3.64 ± 0.07	3.54 ± 0.05	1.13 ± 0.05
24.45 mg/kg	1.05	0	98.85	99/90	0.43 ± 0.02	3.65 ± 0.09	3.55 ± 0.06	1.13 ± 0.05
Negative control	1.08	0	98.92	94/89	0.43 ± 0.03	3.65 ± 0.08	3.55 ± 0.06	1.13 ± 0.05

## Discussion

*M. cordata* is a commonly used topical drug in traditional Asian ethnomedicine and its use has been restricted due to transoral toxicity. Modern research has revealed that its main component, SAN, has a variety of biological activities and is used extensively in animal husbandry as a farming input to provide animal performance. Due to the long-term use in food animals and the potential toxicity risk of SAN, the safety information of MCE as a plant extract that can be added for a long time is of concern. Previous risk assessments of SAN were mostly based on intraperitoneal injections, whereas the most important route for the risk of ingestion of exogenous substances is via the oral route. Therefore, in this study, we systematically evaluated the safety of MCE by performing acute toxicity and long-term oral toxicity studies (subchronic and chronic toxicity) in rodents, while reassessing genotoxicity and developmental toxicity. This is the first systematic report on the safety of MCE after it was developed as a veterinary drug in China. In this report, MCE lacks long-term oral toxicity in rodents as well as mutagenic potential. In conclusion, our study provides important information for the rational use of MCE and subsequent residual safety assessment through general toxicity in rodents as well as genotoxicity assessment.

In acute toxicity studies in SD rats and ICR mice, the LD_50_ of MCE was 1,564.55 and 1,024.33 mg/kg, respectively, which were classified as low toxic substances in the acute toxicity classification of GHS and WHO compounds. The oral acute toxicity results of MCE in rats were similar to those previously obtained with alkaloid extracts of SAN and *Sanguinaria canadensis* L. (Becci et al., 1987). In the subsequent repeated 90-day oral toxicity test, the highest dose of 156.46 mg/kg did not affect body weight gain or other physiological factors for 90 days, and no toxicity-related clinical manifestations were observed during this period. No abnormalities associated with MCE were also found in the hematological, serum biochemical, profiling, and histopathological examinations, and the no observed adverse effect level (NOEAL) of the subchronic toxicity test was determined to be 156.46 mg/kg. In a previous 90-day oral toxicity study of a mixture of quaternary benzo[c]phenanthridine alkaloids of *M. cordata* in rats, 600 mg/kg was considered a safe dose with no significant difference from the negative control (Zdarilova et al., 2008). In addition, in a 90-day feeding trial in pigs, a daily intake of 5 mg/kg of the combined SA/CHE extract was considered safe (Kosina et al., 2004). There may be differences in the proportion of ingredients in the extract as much as possible, but this seems to improve the safety range of MCE. Nevertheless, since the toxicity of a mixture can be influenced by the type and proportion of constituents in it, safety inferences need to be made with caution until the other components of *M. cordata* alkaloid extracts have been subjected to a definitive risk assessment. Similarly, no signs of toxicity associated with MCE were found in further chronic toxicity studies in rats, and the NOEAL was 78.23 mg/kg.

SAN has been reported to form DNA adducts *in vitro* and to increase the level of DNA single-strand breaks in mouse erythrocytes and bone marrow cells after intraperitoneal injection (Stiborová et al., 2002; Ansari et al., 2005; Kaminskyy et al., 2008). In the current study of the genotoxicity of MCE, the number of revertant colonies in all tested strains did not increase at the highest dose of 100 μg/dish. showed agreement with the results of a previous SAN trial in the *E. coli* PQ37 genotoxicity assay (SOS chromosome) (Kevekordes et al., 1999). Similarly, MCE showed a lack of mutagenic potential in the mouse sperm deformation test and micronucleus test. In another 90-day rat study, a mixture of 120 mg SAN and CHE per kg of feed was also shown to lack evidence of genotoxicity (Stiborova et al., 2008). In addition, some studies have shown toxic effects of SAN on oocytes *in vitro* and developmental risk after embryo transfer (Chan, 2011; Chan, 2015), but in the present study, the results of the conventional teratogenic test in rats were not consistent with this. Within the dose range tested, MCE did not exhibit significant maternal damaging effects or fetal developmental toxicity in pregnant rats. In conclusion, the experimental results in this study contradict the previously reported results, and the reasons for this are related to the nature of the SAN itself. Early risk assessments of SAN used almost exclusively intraperitoneal injections, ignoring the interaction between the gastrointestinal tract and SAN. Previous studies tested the stability of SAN and CHE in an artificially simulated gastrointestinal environment and showed that SAN and CHE were stable in an acidic solution, but the average recoveries of SAN and CHE were only 60.53% and 87.89% in an alkaline environment at pH = 8 (Zhang et al., 2017). The mRNA expression of CYP1A1 and CYP1A2 was increased in porcine intestinal epithelial cells after the administration of 5 and 50 μg/ml SAN (Palócz et al., 2019). Current evidence suggests that CYP1A1 and CYP1A2 play an important role in the conversion of SAN to dihydrosanguinarine (DHSA, the first metabolite of SAN metabolic transformation) (Psotová et al., 2006; Deroussent et al., 2010; Sandor et al., 2018) and this is considered to be a possible detoxification process for SANs (Vrba et al., 2004). Degradation and metabolism in the intestine may be one of the reasons for the low bioavailability of SAN. Although DHSA has also recently been reported to have anti-inflammatory (Xiang et al., 2022) and analgesic (Gaona-Tovar et al., 2022) properties, whether it can regulate intestinal flora still needs to be elucidated. Several pharmacokinetic studies explain that SAN and CHE have low gastrointestinal absorption and bioavailability, and are rapidly metabolized *in vivo* (Vecera et al., 2007; Hu et al., 2019; Wu et al., 2020; Zhao et al., 2021). Another toxicity test on the alkaloids of *Chelidonium majus* L. (CAL) yielded similar inferences (Gao et al., 2019). *In vitro* metabolism studies have shown that SAN may be metabolized in the intestine to DHSA, which has been shown to be much less toxic than SAN (Vecera et al., 2007; Vrublova et al., 2008; Sandor et al., 2016). The accumulation coefficients for SAN and DHSA after multiple oral administrations were 1.21 and 1.11, respectively, which seems to explain the low toxicity exhibited by long-term addition of SAN at reasonable doses (Wu et al., 2020).

## Conclusion

We systematically evaluated the general and genetic developmental toxicity of MCE. In this study, MCE was found to exhibit hypotoxic properties in acute exposure to SD rats and ICR mice. The corresponding toxicity risk was also not demonstrated in long-term exposure tests. In addition, the results of systematic mutagenicity and teratogenicity tests showed a lack of evidence of genotoxicity by oral ingestion of MCE at the doses tested. The results of this study provide preliminary and more holistic risk assessment information for MCE in the context of veterinary clinical and human food safety. It is important to note that the results of this study are based on standardized MCE test substances manufactured in GMP production plants. Caution should be exercised when using other *M. cordata* products that are different from MCE, as the difference in toxicity of the mixture depends mainly on the composition and proportions involved.

## Data Availability

The original contributions presented in the study are included in the article/supplementary material, further inquiries can be directed to the corresponding authors.
